# *Coix lacryma-jobi var. ma-yuen* Stapf sprout extract induces cell cycle arrest and apoptosis in human cervical carcinoma cells

**DOI:** 10.1186/s12906-019-2725-z

**Published:** 2019-11-15

**Authors:** Eun Suk Son, Se-Hee Kim, Young Ock Kim, Young Eun Lee, Sun Young Kyung, Sung Hwan Jeong, Yu Jin Kim, Jeong-Woong Park

**Affiliations:** 1 Department of Allergy, Pulmonary and Critical Care Medicine, Gachon University, Gil Medical Center, Incheon, 21565 Republic of Korea; 2 Gachon Medical Research Institute, Gachon University Gil Medical Center, Incheon, 21565 Republic of Korea; 3 Department of Bio-Environmental Chemistry, College of Agriculture and Life Sciences, Chungnam National University, Daejeon, 34134 Republic of Korea; 4 Division of Pulmonology, Department of Internal Medicine, Korea University Ansan Hospital, Gyeonggi-do, 15355 Republic of Korea

**Keywords:** Apoptosis, Cell cycle arrest, Cervical cancer, *Coix lacryma-jobi* sprout extract

## Abstract

**Background:**

Cervical cancer is the second-leading cause of cancer-related mortality in females. *Coix lacryma-jobi* L. var. ma-yuen (Rom.Caill.) Stapf ex Hook. f. is the most widely recognized medicinal herb for its remedial effects against inflammation, endocrine system dysfunctions, warts, chapped skin, rheumatism, and neuralgia and is also a nourishing food.

**Methods:**

To investigate the activity of *Coix lacryma-jobi* sprout extract (CLSE) on cell proliferation in human cervical cancer HeLa cells, we conducted a Cell Counting Kit-8 (CCK-8) assay. Flow-cytometric analysis and western blot analysis were performed to verify the effect of CLSE on the regulation of the cell cycle and apoptosis in HeLa cells.

**Results:**

We observed that CLSE significantly inhibited cell proliferation. Furthermore, CLSE dose-dependently promoted cell cycle arrest at the sub-G1/ S phase in HeLa cells, as detected by bromodeoxyuridine (BrdU) staining. The cell-cycle-arrest effects of CLSE in HeLa cells were associated with downregulation of cyclin D1 and cyclin-dependent kinases (CDKs) 2, 4, and 6. Moreover, CLSE induced apoptosis, as determined by flow-cytometric analysis and nuclear DNA fragmentation with Annexin V/propidium iodide (PI) and 4′6′-diamidino-2-phenylindole (DAPI) staining. Induction of apoptosis by CLSE was involved in inhibition of the antiapoptotic protein B-cell lymphoma 2 (Bcl-2) and upregulation of the apoptotic proteins p53, cleaved poly (ADP-ribose) polymerase (PARP), cleaved caspase-3, and cleaved caspase-8. Finally, we observed that CLSE inactivated the phosphoinositide 3-kinase (PI3K) and protein kinase B (AKT) pathways.

**Conclusions:**

CLSE causes cell cycle arrest and apoptotic cell death through inactivation of the PI3K/AKT pathway in HeLa cells, suggesting it is a viable therapeutic agent for cervical cancer owing to its anticancer effects.

## Background

Cervical cancer is the second-leading cause of cancer-related mortality in females [1, 2]. Athough the pathological process of cervical cancer is still ambiguous, nearly all cases of cervical cancer are caused by human papillomavirus (HPV) infection [3, 4]. HPV can activate the PI3K/AKT/mTOR pathways and disturb the cellular mechanisms for growth control [5, 6]. Although new chemotherapeutic agents for the most common cancer have developed over the past few decades, the number of cancer-related deaths remains high due to metastasis and drug resistance [7]. Therefore, the development of chemopreventive or chemotherapeutic agents against cervical carcinoma is crucial to reduce the incidence, mortality, and prevalence of this disease [8].

The regulation of cell cycle arrest and apoptotic cell death is an important feature of anticancer agents [9, 10]. The cell cycle is responsible for cell duplication, and cell cycle progression is checked at checkpoints in the G1/S, S, and G2/M phases [11, 12]. These cell cycle checkpoints are triggered by DNA damage and misaligned chromosomes at the mitotic spindle [13]. Deregulations of apoptotic cell death and the cell cycle is associated with aberrant cell proliferation and cancer [14]. Therefore, treatment of tumor cells usually results in the breakdown of the cell cycle machinery, leading to the inhibition of cell proliferation and induction of apoptosis [15].

Several natural products have been demonstrated to have antitumor effects with few side effects. Specifically, these products can kill cancer cells by modulating apoptosis [16, 17]. In recent years, many studies have investigated the potential anticancer properties of natural products that are considered to be non-toxic and thus may have fewer side effects compared with synthetic compounds [18–21].

*Coix lacryma-jobi var. ma-yuen* (Rom.Caill.) Stapf ex Hook. f. is a tropical plant of the family Poaceae and is native to Southeast Asia, ranging from India through Malaysia to China [22]. It is now widely grown in other places. *Coix* has high protein content compared with rice and serves as a rice alternative. Previous studies demonstrated that *Coix* shows apoptotic and antiproliferative effects against human breast cancer, lung cancer, hepatocellular carcinoma cells, colon cancer cells, and histolytic lymphoma [23–28]. *Coix* sprouts are obtained from seeds during sprouting. Sprouting is the practice of germinating seeds to be eaten raw or cooked. Thus, germination can lead to the development of functional foods that have a positive effect in humans and can help maintain health [29].

Over the past few decades, *Coix* seeds have been studied extensively, and anticancer mechanisms, including cell cycle arrest and apoptosis, have been discovered. However, the effects of *Coix lacryma-jobi* sprout extract (CLSE) on anticancer mechanisms remain elusive. In this study, our goal was to evaluate the antitumor activities of CLSE in human cervical carcinoma cells.

## Methods

### Cell culture and reagents

Human cervical cancer HeLa cells were purchased from the Korean Cell Line Bank (Seoul, South Korea). HeLa cells were maintained in RPMI 1640 (Gibco Cell Culture, Carlsbad, CA, USA) with 1% penicillin (Gibco), 1% streptomycin (Gibco), and 10% fetal bovine serum (Gibco) at 37 °C in a humidified atmosphere of 5% CO_2_.

CLSE was extracted at the Herbal Crop Research Institute, Rural Development Administration (Chungbuk, South Korea) [30]. SC79 was purchased from Sigma-Aldrich (St. Louis, MO, USA).

### Cell proliferation assay

Cell viability was assessed using the cell counting kit (CCK)-8 (Dojindo Molecular Technologies, Inc., Rockville, MD, USA). HeLa cells were plated in a 96-well plate and treated with CLSE (0, 125, 250, 500, or 1000 μg/mL) for 24–72 h. Then, cells were incubated with CCK-8 at 37 °C for 2 h. The absorbance was measured at 550 nm.

### Flow-cytometric analysis of the cell cycle

After cells were treated with CLSE (0, 250, 500, or 1000 μg/mL) for 72 h, cells were harvested and then washed with phosphate-buffered saline (PBS). Cell cycle progression was induced by following the manufacturer’s instructions for the FITC bromodeoxyuridine (BrdU) Flow Kit (BD Bioscience, San Diego, CA, USA). The cell cycle was monitored by DNA content using a FACSCalibur cell analyzer (BD Bioscience) after the cells were stained to evaluate the sub-G1, G0/G1, S, and G2/M phase rates.

### Flow-cytometric analysis of cell apoptosis

After treatment with CLSE for 72 h, HeLa cells were resuspended in 100 μL binding buffer and stained with FITC-annexin and propidium iodide (PI) (BD Bioscience). Then, 400 μL binding buffer was added, and the cells were analyzed using a FACSCalibur system. All tests were performed three times independently.

### 4′6′-Diamidino-2-phenylindole (DAPI) staining

In brief, HeLa cells were seeded in a 4-well cell culture slide and treated with CLSE (0–1000 μg/mL) for 48 h. The cell monolayer was fixed with 4% paraformaldehyde. After permeabilization with PBS containing 0.1% Triton X-100, the cells were incubated with 1 μg/mL DAPI. Finally, the fragmented nuclei and condensed chromatin were observed under a confocal microscope (Zeiss, Oberkochen, Germany).

### Western blot analysis

After treatment with CLSE for 24–72 h, HeLa cells were lysed with radioimmunoprecipitation assay (RIPA) buffer (50 mM Tris, 150 mM NaCl, 2 mM EDTA, and 1% NP-40) for 5 min at 4 °C. The primary antibodies for cyclin D1 and cyclin-dependent kinase (CDK) 2, CDK4, CDK6, GAPDH, p18^INK4c^, p21^Waf1/Cip1^, p27^Kip1^, p53, poly (ADP-ribose) polymerase (PARP), cleaved caspase-3, cleaved capase-8, Bcl-2, Bcl-2 associated X (Bax), PI3K, phospho-PI3K, AKT, and phospho-AKT (Ser 473) were obtained from Cell Signaling Technology (Danvers, MA, USA). Glyceraldehyde 3-phosphate dehydrogenase (GAPDH) was used as the loading control.

### Statistical analysis

The data values were analyzed as the means ± standard deviation of three independent experiments. One-way ANOVA followed by Tukey’s test was done for data comparison among groups. *p < 0.05, **p < 0.01, and ***p < 0.001 were considered to indicate statistical significance.

## Results

### Effects of CLSE on HeLa cell viability

To examine whether CLSE regulates the viability of HeLa cells, cells were treated with CLSE (0–1000 μg/mL) for 24–72 h, and the cell viability was evaluated using the CCK-8 assay.

**Fig. 1:**
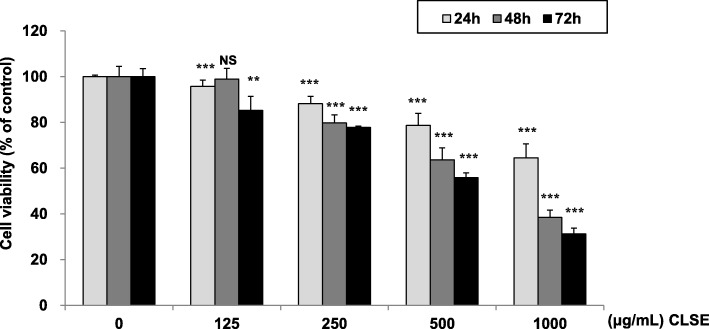
Cytotoxic effect of *Coix lacryma-jobi* sprout extract (CLSE) on HeLa cells. Cell viability was assessed by the Cell Counting Kit-8 after treatment with different concentrations of CLSE (0–1000 μg/mL) for 24–72 h. **p < 0.01 and ***p < 0.001 versus control group. NS, not significant. All data values are expressed as the mean ± standard deviation (*n* = 5)

As expected, the cell viability in CLSE-treated cells decreased in a time- and dose-dependent manner (Fig. 1). The half-maximal inhibitory concentration (IC_50_) value of CLSE was 1723, 720.8, and 580.2 μg/mL at 24, 48, and 72 h, respectively.

### Effects of CLSE on cell cycle distribution in HeLa cells

**Fig. 2:**
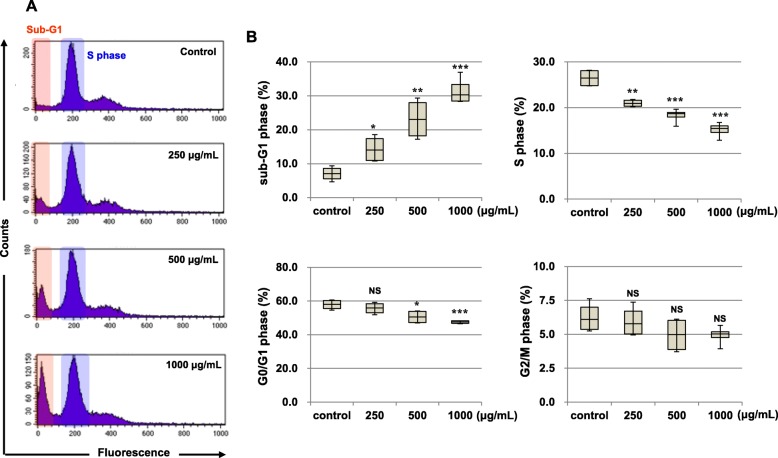
Effects of *Coix lacryma-jobi* sprout extract (CLSE) on HeLa cell cycle progression. **a, b** HeLa cells were stained with BrdU after treatment with CLSE (0–1000 μg/mL) for 72 h, and cell cycle distribution was monitored using flow-cytometric analysis. Dose–response graphs demonstrating the percentage of sub-G1, S, G0/G1, and G2/M (B) phases from the data displayed in panel (**a**). *p < 0.05, **p < 0.01, and ***p < 0.001 versus control cells. NS, not significant. Data values are expressed as the mean ± standard deviation (*n* = 4)

To examine whether CLSE inhibits HeLa cell growth via cell cycle regulation, we investigated the cell cycle distribution using flow cytometry. CLSE (0, 250, 500, and 1000 μg/mL) decreased the percentage of S phase cells from 26.44 to 20.95%, 18.22, and 15.13%, respectively (Fig. 2a and b). No changes in the G2/M phase population were evident after CLSE treatment, and the G0/G1 phase population was decreased (Fig. 2b). Rather, the proportion of cells with sub-G1 phase was elevated (Fig. 2a and b). The proportion of HeLa cells in the sub-G1 phase was increased to 7.0–31.5% by treatment with CLSE. The increase in the sub-G1 population indicated that CLSE induced HeLa cell death, because the sub-G1 population represents apoptotic cells. These results suggest that both S phase arrest and apoptotic cell death were increased by CLSE treatment.

**Fig. 3:**
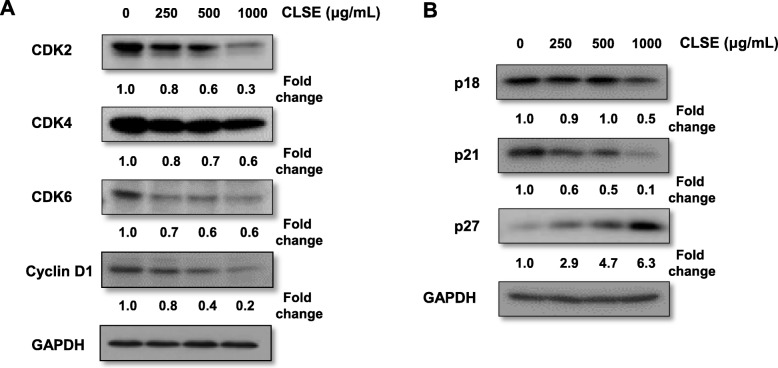
Effects of *Coix lacryma-jobi* sprout extract (CLSE) on the modulation of cell cycle regulatory proteins. **a, b** After HeLa cells were treated with CLSE for 72 h, the expression of cell cycle regulatory proteins (CDK2, CDK4, CDK6, cyclin D1, p18, p21, and p27) was examined using western blot analysis

To further examine the biochemical events regulated by CLSE, we examined the expression levels of cell cycle regulatory proteins. CLSE dose-dependently reduced the protein levels of cyclin D1, CDK2, CDK4, and CDK6. These proteins regulate G1/S phase progression, and CLSE significantly inhibited their expression in HeLa cells (Fig. 3a). To determine the regulators involved in cell cycle arrest, the cyclin-dependent kinase inhibitors p18^INK4c^, p21^Waf1/Cip1^, and p27^Kip1^, which regulate cell cycle progression, were investigated using western blot analysis. CLSE treatment showed a markedly increased (6.3-fold) p27^Kip1^ protein level, whereas the protein levels of p18^INK4c^ (2.0-fold) and p21^Waf1/Cip1^ (10.0-fold) decreased (Fig. 3b). These results suggest that p27^Kip1^ caused cell cycle arrest in CLSE-treated HeLa cells.

### Effects of CLSE on apoptosis in HeLa cells

**Fig. 4:**
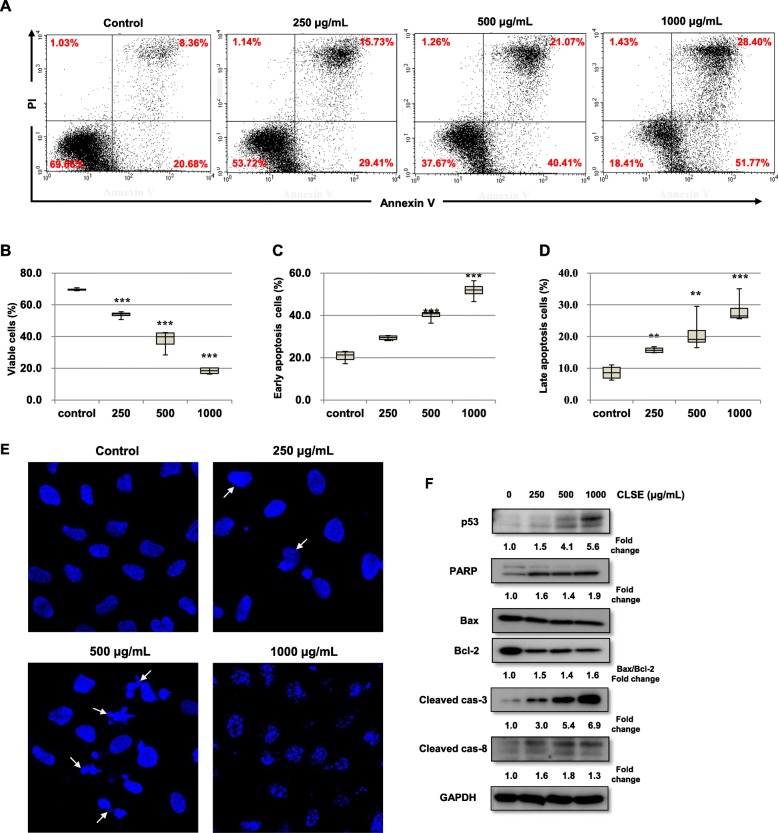
Effects of *Coix lacryma-jobi* sprout extract (CLSE) on HeLa cell apoptosis. **a–d** HeLa cells were stained with FITC-annexin V/propidium iodide (PI) after treatment with various concentrations of CLSE (0–1000 μg/mL) for 72 h, and cell populations and the extent of apoptosis were measured by flow-cytometric analysis. **a** Representative cell apoptosis dot plots for FITC-annexin V/PI staining in CLSE-treated cells. **b-d** Quantification of viable cells (FITC-annexin V^−^/PI^−^), early-apoptotic cells (FITC-annexin V^+^/PI^−^), and late-apoptotic cells (FITC-annexin V^+^/PI^+^) using four independent cell populations. **e** HeLa cells were stained with DAPI (1 μg/mL) after treatment with CLSE (1–1000 μg/mL) for 48 h and observed under a confocal microscope. Arrow, apoptotic cells (magnification: × 400). **f** HeLa cells were treated with CLSE (1–1000 μg/mL) for 72 h, and apoptosis-related proteins (p53, PARP, Bax, Bcl-2, cleaved caspase-3, and cleaved caspase-8) were examined using western blot analysis. **p < 0.01 and ***p < 0.001 versus control group. All data values are expressed as the mean ± standard deviation (n = 4)

To verify whether CLSE inhibits HeLa cell growth via apoptosis, CLSE-treated HeLa cells were examined using flow cytometry. Annexin V is a phospholipid-binding protein with high affinity for phosphatidylserine (PS). Apoptotic cells show the redistribution of membrane PS from the inner to the outer leaflet of the plasma membrane. Representative dot plots illustrating apoptotic status are shown in Fig. 4a. Treatment with CLSE (0, 250, 500, and 1000 μg/mL) dose-dependently decreased the percentage of viable cells (Annexin V^−^/PI^−^) (Fig. 4b). The proportion of early-apoptotic cells (Annexin V^+^/PI^−^) treated with CLSE increased from 20.68 to 29.41%, 40.01, and 51.77%, respectively (Fig. 4c). Furthermore, the proportion of late-apoptotic cells (Annexin V^+^/PI^+^) treated with CLSE increased from 8.63 to 15.73%, 21.07 and 28.40%, respectively (Fig. 4d).

Apoptotic cell death manifests in features such as chromatin condensation, cell shrinkage, and nuclear fragmentation. To observe changes within the nucleus during apoptosis of CLSE-treated HeLa cells, we stained CLSE-treated HeLa cells with DAPI. As shown in Fig. 4e, cells treated with CLSE exhibited dose-dependent DNA fragmentation and bright nuclear condensation compared with the control cells.

To investigate whether CLSE-induced apoptosis is mediated by caspases in HeLa cells, we checked the expression levels of the pro-apoptotic proteins PARP, Bax, and cleaved caspase-3 and the antiapoptotic protein Bcl-2 in CLSE-treated HeLa cells. As shown in Fig. 4f, CLSE decreased Bcl-2 and increased cleaved PARP and cleaved caspase-3 in HeLa cells. There were no changes in the expression level of Bax. Collectively, these results suggest that increased Bax/Bcl-2 ratio might be involved in CLSE-induced apoptosis.

### Effects of CLSE on the PI3K/AKT pathway

**Fig. 5:**
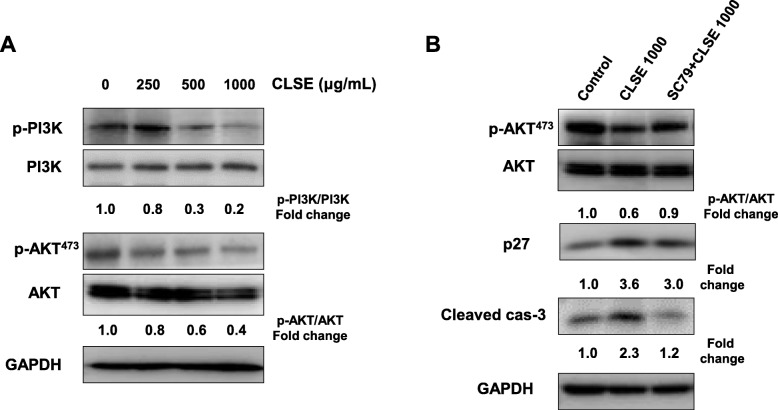
Effects of *Coix lacryma-jobi* sprout extract (CLSE) on signaling pathways in HeLa cells. HeLa cells were treated with CLSE (1–1000 μg/mL) (**a**) and/or SC79 (5 μg/mL) (**b**) for 24–72 h, and apoptosis-related proteins (p-PI3K, PI3K, p-AKT, AKT, p27, and cleaved caspase-3) were detected using western blot analysis

To identify which signaling pathways are modulated by CLSE, we investigated the expression of PI3K and AKT using western blot analysis. As shown in Fig. 5a, CLSE-treated HeLa cells reduced the phosphorylation of PI3K (5.0-fold) and AKT (2.5-fold) relative to the control group (Fig. 5a). We further confirmed that the activation of AKT was repressed by CLSE using the AKT activator SC79. As expected, treatment with SC79 recovered CLSE’s inhibition of the AKT signaling pathway (Fig. 5b). Furthermore, SC79 lowered the protein levels of p27 and cleaved caspase-3 despite the presence of CLSE. Therefore, our results suggest that CLSE induces HeLa cell cycle arrest and apoptosis through inactivation of the PI3K/AKT pathway.

## Discussion

Cervical cancer is the fourth most common cancer in women worldwide and remains a major global health problem [31]. Currently, all applicable anticancer drugs have severe side effects and resistance [32]. Thus, alternative medicines have appeared as new anticancer agents with fewer side effects [33]. Medicinal plants are important sources in the development of anticancer drugs [34].

Recently, a number of studies on the anticancer effects of *Coix* have been reported. Manosroi et al. demonstrated that the hull of *Coix* extract has an antiproliferative effect on colon cancer cells [28]. In addition, *Coix* seed oil injection dulls the pain of cancer patients, and *Coix* seed oil combined with Norcantharidin reduces hepatocellular carcinoma growth and induces apoptosis [35, 36]. We also reported that *Coix* sprout extract suppresses invasion and migration of colon cancer cells and tube formation of human endothelial cells in low-oxygen conditions [30]. Accordingly, recent studies provide a scientific basis for our study of anticancer effects of *Coix* in human cervical cancer cells.

Additionally, recent studies have focused on identifying the active components of *Coix* and their mechanisms. *Coix* contains a large number of lipopolysaccharides, such as stearic acid, palmitic acid, oleic acid, octadecadienoic acid, and linoleic acid. Lu et al. suggest that the polysaccharide fraction of *Coix* seed is able to inhibit A549 cell proliferation and induce apoptosis [37]. Neutral lipids isolated from *Coix* inhibit the proliferation of pancreatic cancer cells [38]. Five compounds (coixlactam, methyl dioxindole-3-acetate, and coixspirolactam A, B, and C) isolated from *Coix* bran inhibit the proliferation of colon and lung cancer cells [26]. Other edible sprouts contain many bioactive phytochemicals, such as isothiocyanates, phenolic acids, flavonoids, and polyphenols, which can perform anticancer functions [39]. We believe that *Coix* sprout extract will be found to contain similar ingredients as the mature *Coix* and other generally edible sprouts. For this reason, we will endeavor in our future studies to isolate the active components of *Coix* sprout extract, which may include coixlactam, neutral lipids, and phytochemicals, and find their possible roles in the anticancer functions of CLSE.

Even though recent studies have demonstrated that *Coix* has antitumor activities in many cancer cells, studies on the mechanisms of *Coix* are relatively scarce. In our previous study, a sprout extract of *Coix* inhibited the metastasis of colon cancer cells; CLSE showed inhibitory effects on invasion, migration, adhesion, and wound healing of colon cancer cells [30]. However, the signaling pathways regulated by CLSE in cervical cancer have not been reported. Thus, in this study, we examined whether CLSE exhibited anticancer activity through cell cycle arrest and apoptotic cell death in cervical cancer cells.

Cell cycle progression is an important mechanism for homeostatic maintenance in normal cells [40]. However, the deregulation of cell cycle progression is a common characteristic of cancer cells [10]. Therefore, the regulation of the cell cycle in cancer cells is the basic question to be addressed prior to clinical studies for anticancer drug development [41]. In this study, CLSE inhibited HeLa cell proliferation, significantly decreased the percentage of S phase cells, and effectively increased the percentage of sub-G1 phase cells. However, CLSE did not promote the progression of the cell cycle to the G0/G1 and G2/M phases. It has been reported that the antitumor activities of lung cancer cells mediated by a polysaccharide fraction of *Coix* seed were associated with S phase arrest and apoptosis. Although many studies have focused on G1 or G2 phases as DNA damage checkpoints, CLSE may specifically affect the proportion of S phase cells without regulating the G0/G1 or G2/M phases [42, 43].

Cell cycle regulation involves changes in the expression of three major classes of regulators: cyclins, CDKs, and cyclin-dependent kinase inhibitors (CDKIs) [11]. While the cyclin D/CDK4/CDK6 and cyclin E/CDK2 complexes modulate S-phase entry, these complexes are regulated by CDKIs [44]. CDKIs such as p21^waf1/Cip1^ and p27^Kip1^ are tumor suppressors that inhibit cell cycle progression by association with active cyclin/CDK complexes [7]. p21^CIP1^ is required for cyclin D/CDK4 assembly early in the G1 phase [45], whereas p27^KIP1^ is a critical modulator of the G1/S transition. Furthermore, p21^CIP1^ −/− mice do not develop spontaneous tumors, whereas p27^KIP1^−/− mice spontaneously develop both lung and pituitary adenomas [46]. Therefore, p27^KIP1^ is a critical tumor suppressor that regulates the cell cycle. On this basis, we showed that CLSE inhibited the protein levels of cyclin D and CDKs (CDK2, − 4, and − 6) and significantly increased p27^KIP1^ expression level in HeLa cells. Collectively, our results suggest that CLSE regulates various molecules involved in S phase progression.

The three pathways leading to cell death are autophagy, apoptosis, and necrosis [47]. Most current anticancer drugs induce apoptosis of cancer cells. Apoptotic cell death has been suggested as an important mechanism for cancer chemotherapy and chemoprevention. Thus, agents that selectively induce apoptosis in cancer cells may be useful for cancer treatment. In this study, we found that CLSE induced the characteristic morphology of apoptosis, such as nuclear deformity, cell shrinkage, and nuclear fragmentation, in HeLa cells, and significantly induced dose-dependent increases in early- and late-apoptotic cells.

The regulation of Bax and Bcl-2 in apoptotic cell death can be explained by their respective protein levels and the ratio of two proteins [48]. Generally, the Bax/Bcl-2 ratio is increased by high mitochondrial membrane permeability, which results in cytochrome C release from the mitochondria and the activation of downstream caspases [49]. Cleaved caspases are active forms and are considered robust signals of apoptosis [50]. Additionally, PARP and p53 are important factors for several important biological functions, such as DNA repair, cell cycle regulation, and apoptosis [16]. Thus, the upregulation of PARP cleavage and p53 by caspases is considered to be a hallmark of apoptosis [51]. Here, we found that the expression level of Bax did not change but the Bax/Bcl-2 ratio was increased in CLSE-treated HeLa cells. We also observed that CLSE induced the protein levels of p53, cleaved PARP, cleaved caspase-3, and cleaved caspase-8 in HeLa cells. Although some CLSE concentration levels at IC_50_ appear too high to positively correlate the compound with the cancer cell cytotoxicity, results from other assays including FACS, DNA fragmentation, and western blot clearly demonstrate the anticancer effect of the CLSE.

PI3K/AKT signaling is involved in multiple cellular processes, such as survival and the proliferation of several cell types [52, 53]. In addition, PI3K/AKT signal transduction is able to promote various cancers because this pathway modulates multiple cancer-related processes, such as proliferation, apoptosis, and metastasis, by regulating a number of downstream transcription factors [54, 55]. Many studies have reported that PI3K/AKT signal transduction induces tumorigenesis, proliferation, inhibition of apoptosis, and chemoresistance [53]. In a previous study, we demonstrated that CLSE inhibited the migratory properties of colon cancer cells through repression of AKT and ERK1/2 phosphorylation in low-oxygen conditions. Our results also confirmed that CLSE downregulated the phosphorylation of PI3K and AKT; furthermore, AKT activation by SC79 decreased the expression of p27 and cleaved caspase-3 proteins induced by CLSE in HeLa cells. Collectively, these data suggest that CLSE promotes cell cycle arrest and apoptosis through inhibition of AKT phosphorylation.

Although our research has reported anticancer effects of *Coix* sprout extract, more research on the stability and clinical effect of herbal medicines containing *Coix* is required. In the future, we will reinforce our findings by utilizing an in vivo mouse model and chemical analysis of active components of *Coix* sprout extract.

## Conclusion

In this paper, CLSE showed antitumor activities in human cervical cancer cells. Specifically, CLSE treatment resulted in cell cycle arrest by reducing the proportion of S phase cells, decreasing the expression of cyclin D and CDKs (CDK2, − 4, and − 6), elevating p27^KIP1^ expression, and inducing apoptosis by upregulating p53, cleaved PARP, cleaved caspase-3, and cleaved caspase-8 through impairment of the PI3K/AKT pathway. Consequently, CLSE may be an effective therapeutic agent for cervical cancer, as it may induce cell cycle arrest and apoptosis via inactivation of the PI3K/AKT pathways. In the future, we will further study the effectiveness of the antitumor activities of CLSE in cervical cancer.

## Data Availability

The data that support the findings of this study are available from the corresponding author upon reasonable request.

## Abbreviations

CCK-8: Cell Counting Kit-8
CDK: Cyclin-dependent kinase
CDKIs: Cyclin-dependent kinase inhibitors
CLSE: *Coix lacryma-jobi var. ma-yuen* Stapf sprout extract
HPV: Human papillomavirus
PARP: Poly (ADP-ribose) polymerase
PBS: Phosphate-buffered saline
PS: Phosphatidylserine
